# Supplementary material for: Engineering Herbicide-Tolerance Rice Expressing an Acetohydroxyacid Synthase with a Single Amino Acid Deletion
Source: Int J Mol Sci. 2020 Feb 13;21(4):1265. doi: 10.3390/ijms21041265 (PMC7072996; doi:10.3390/ijms21041265)
Supplement: Supplementary file 1 [file ijms-21-01265-s001.zip › ijms-715169-revised-suppl/Figure S1.doc]

Figure S1. The *ahas* relative mRNA level in rice leaf at maturity stage. The lines had herbicide tolerance. The Xiushui134 without herbicides application were used as control. Error bars represented errors from multiple measurements.
